# Post-secretional activation of Protease IV by quorum sensing in *Pseudomonas aeruginosa*

**DOI:** 10.1038/s41598-017-03733-6

**Published:** 2017-06-30

**Authors:** Jungmin Oh, Xi-Hui Li, Soo-Kyong Kim, Joon-Hee Lee

**Affiliations:** Department of Pharmacy, College of Pharmacy, Pusan National University, Busan, 609-735 South Korea

## Abstract

Protease IV (PIV), a key virulence factor of *Pseudomonas aeruginosa* is a secreted lysyl-endopeptidase whose expression is induced by quorum sensing (QS). We found that PIV expressed in QS mutant has severe reduction of activity in culture supernatant (CS), even though it is overexpressed to high level. PIV purified from the QS mutant (M-PIV) had much lower activity than the PIV purified from wild type (P-PIV). We found that the propeptide cleaved from prepro-PIV was co-purified with M-PIV, but never with P-PIV. Since the activity of M-PIV was restored by adding the CS of QS-positive and PIV-deficient strain, we hypothesized that the propeptide binds to and inhibits PIV, and is degraded to activate PIV by a QS-dependent factor. In fact, the CS of the QS-positive and PIV-deficient strain was able to degrade the propeptide. Since the responsible factor should be a QS-dependently expressed extracellular protease, we tested QS-dependent proteases of *P*. *aeruginosa* and found that LasB (elastase) can degrade the propeptide and activate M-PIV. We purified the propeptide of PIV and confirmed that the propeptide can bind to and inhibit PIV. We suggest that PIV is post-secretionally activated through the extracellular degradation of the propeptide by LasB, a QS-dependent protease.

## Introduction

*Pseudomonas aeruginosa* is a Gram negative opportunistic human pathogen causing infections to cornea, burn wounds, and urethra, and severely aggravating several diseases, such as cystic fibrosis and pneumonia^1, 2^. The virulence factors of *P*. *aeruginosa* include proteases, toxins, phenazines, and pyocyanin that play crucial roles in infection^3, 4^. Many of these virulence factors are expressed under the control of quorum sensing (QS), a bacterial cell to cell communication system that regulates a large number of genes in a cell density-dependent manner^5^. In QS response of *P*. *aeruginosa*, small diffusible molecules such as acyl-homoserine lactones (acyl-HSL) and PQS (*Pseudomonas* quinolone signal; 2-heptyl-3-3hydroxy-4-quinolonme) are used for signaling. acyl-HSLs are generally considered as principal QS signals and three signal synthase-receptor systems, LasI-R, RhlI-R, and QscR constitute the entire acyl-HSL QS circuit^6, 7^. The signal-receptor complexes transcriptionally regulate a large number of target genes including virulence factors^5, 7, 8^. Extracellular proteases have been considered very important virulence factors of pathogens and *P*. *aeruginosa* produces many extracellular proteases, such as elastase (LasB, pseudolysin), LasA (staphylolysin), LasD (staphylolysin), alkaline protease (AprA, aeruginolysin), small protease (PASP), large extracellular protease (LepA), and protease IV (PIV)^2, 9–14^. Many of them, such as LasB, LasA, AprA, and PIV are under the transcriptional control of QS^7, 11^. Thus the QS mutant that is incapable of producing acyl-HSLs is much less virulent with tiny production of extracellular proteases^11^.

PIV is a lysine-specific endoprotease encoded in the *piv* gene that is highly induced by acyl-HSL in *P*. *aeruginosa* and a major virulence factor for the *Pseudomonas* corneal and lung infections^2, 15, 16^. It also works as a virulence factor to various invertebrates, such as *Tenebrio molitor* (insect), *Caenorhabaditis elegans* (nematode), and *Artemia salina* (aquatic crustacean)^11^. Since a mutation on *piv* gene causes *P*. *aeruginosa* to be much less virulent to these invertebrates as QS mutations and parenteral inject of purified PIV effectively kills insects without other factors^7, 11^, it was expected that the artificial expression of PIV by non-QS promoter (eg, arabinose-inducible promoter) should restore the virulence of the QS mutant to insect. However, we found that QS mutant overexpressing PIV by arabinose-inducible promoter was not able to kill insect, even though it produced high levels of normal PIV. Surprisingly, the PIV overexpressed in QS mutant was not very active even when it was purified. However, the addition of the culture supernatant from QS-positive cells restored the activity of the PIV purified from QS mutant. These results strongly implied that an unrevealed mechanism of QS system additionally regulates the PIV activity even after secretion. In this study, we elucidated this post-secretional QS regulation on the PIV activity.

PIV is initially expressed as 48 kDa full length in cytoplasm (prepro-PIV), but processed twice from N-terminus during secretion^17^. First processing removes its signal peptide to produce 45 kDa (pro-PIV) that is further processed to cleave the propeptide off, making 26 kDa mature PIV (Fig. S1). In this study, we detected a co-purified band with PIV when we purified PIV from the QS mutant strain, whereas it was never co-purified from wild type. We found that it is the propeptide cleaved from pro-PIV and it binds to PIV to inhibit the PIV activity. In addition, we found this inhibition was relieved by LasB, another QS-dependent extracellular protease that degraded the propeptide. Through this mechanism, QS may coordinate the activation of PIV even after secretion.

## Results

### PIV expressed in QS mutant has much lower activity than PIV expressed in wild type

**Figure 1:**
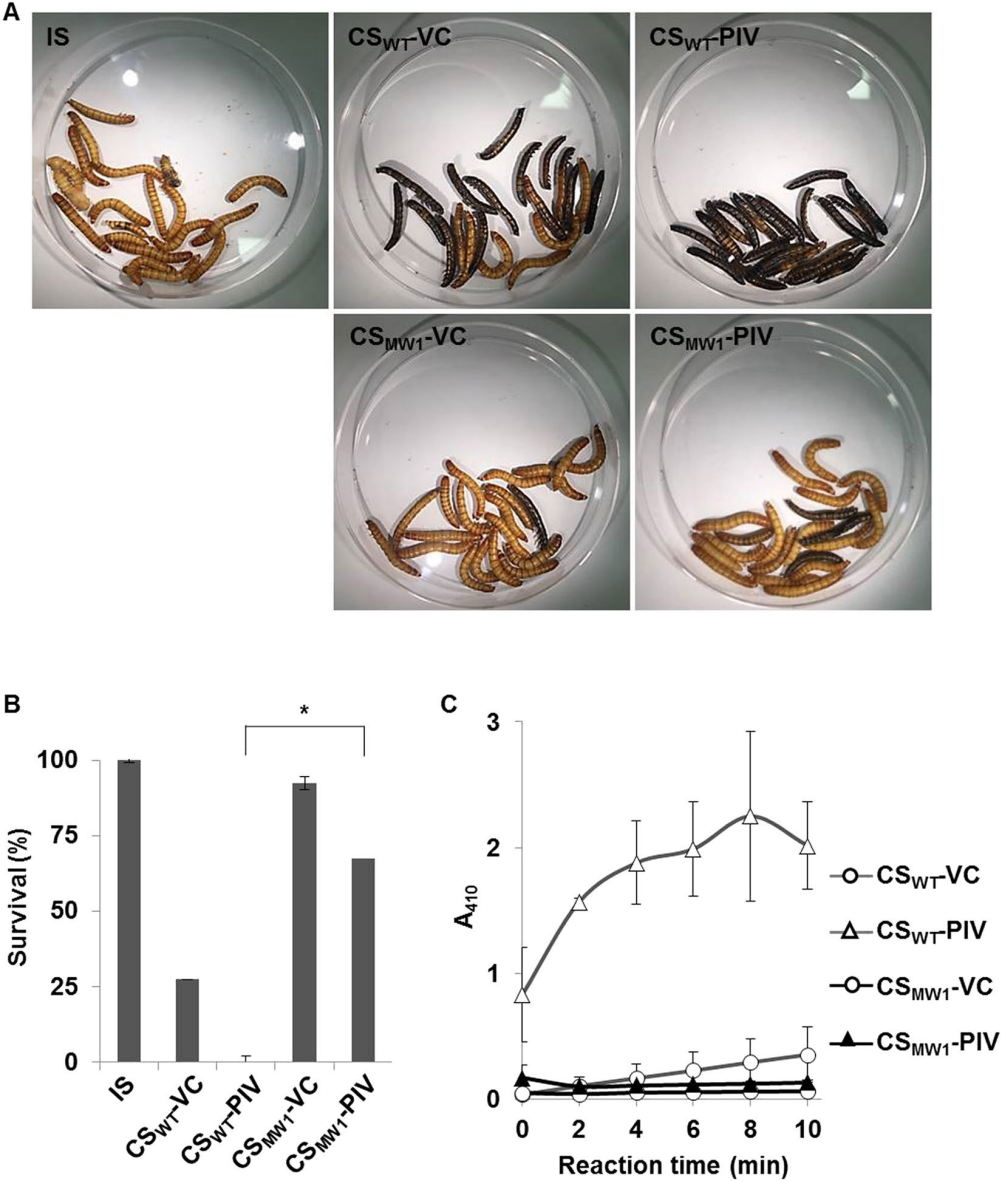
Reduced activity of PIV expressed in QS mutant. The CSs prepared from the PIV-overexpressing (pSP301-harboring) PAO1 (CS_WT_-PIV) or MW1 (CS_MW1_-PIV) were injected into *T*. *moliter* larvae, and the death with melanization was observed for 4 days (**A**) and quantified (**B**). The CSs were concentrated 10 times before injection and 5 μl of CS was injected. Insect saline (IS) was injected for injection control and CSs prepared from pJN105-harboring PAO1 or MW1 (CS_WT_-VC) or MW1 (CS_MW1_-VC) were injected for vector control (VC). *p < 0.01. (**C**), the activity of PIV in the CSs was measured by chromogenic substance. Absorbance at 410 nm (A_410_) was measured every 2 min for 10 min at 37 °C.

Since purified PIV alone can kill *T*. *molitor* larvae efficiently^11^, we attempted to restore the virulence of QS mutant by artificially expressing PIV using arabinose-inducible promoter. PIV was overexpressed on pSP301 in PAO1 (wild type) and MW1 (a QS mutant that does not produce acyl-HSL, Table S1), respectively and their culture supernatants (CSs) were prepared. When the CSs were injected into *T*. *molitor* larvae, the CS from PIV-overexpressing PAO1 (CS_WT_-PIV) strongly killed *T*. *molitor* larva, whereas the CS from PIV-overexpressing MW1 (CS_MW1_-PIV) showed much reduced virulence to *T*. *molitor* larvae (Fig. 1A,B). When the activity of PIV in each CS was specifically measured using chromogenic substrate, PIV in the CS of MW1 was much less active than PIV in the CS of PAO1 (Fig. 1C). These unexpected results strongly implied the existence of additional regulation by QS system other than transcriptional regulation. We confirmed that the PIV expression levels were not different between CS_WT_-PIV and CS_MW1_-PIV (Fig. S2A). The content of PIV in total proteins in CS_WT_-PIV and CS_MW1_-PIV was 48.3% and 48.0%, respectively (Fig. S2B).

### The reduced activity of PIV is due to inhibition by other QS-dependent factor, not by internal modification of PIV

**Figure 2:**
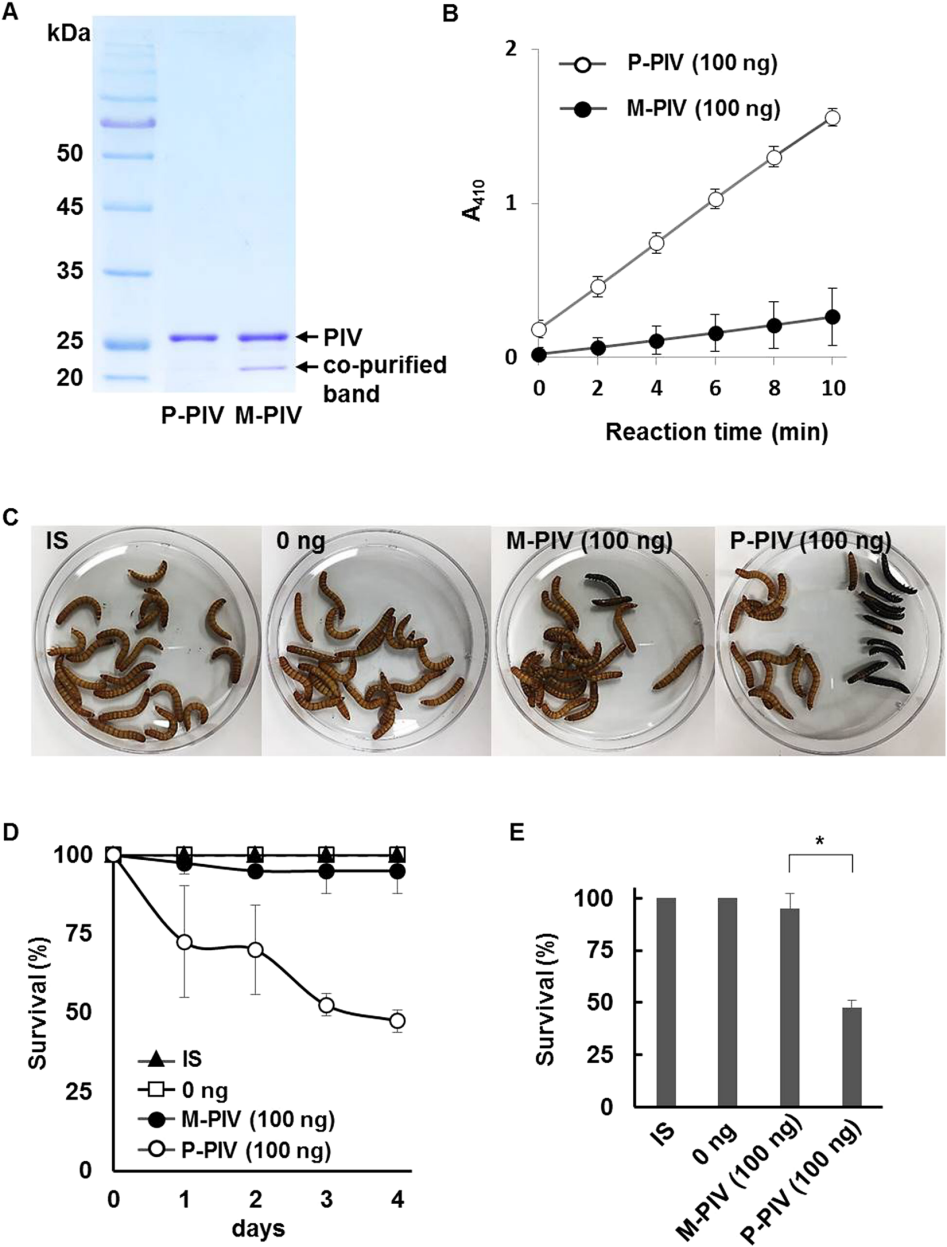
Reduced activity of purified PIV from QS mutant. PIV was purified from PAO-T7 (P-PIV) and PAO-T7-MW1 (M-PIV) and the purity was confirmed in SDS-PAGE (**A**). PIV bands at 26 kDa are indicated. (**B**) the activity of P-PIV and M-PIV was measured at 37 °C using chromogenic substance. Same amount of M-PIV and P-PIV (100 ng) were injected to *T*. *moliter* larvae and death with melanization was observed (**C**). Same volume of insect saline (IS) and protein storage buffer (0 ng) were injected as controls. The survival along with days (**D**) and at day 4 (**E**) was quantified. *p < 0.01.

Because there is a large difference in protein profile between CS_WT_-PIV and CS_MW1_-PIV (Fig. S2A), any factor that exists only in one of the two CSs could affect PIV activity. To exclude other factors, PIV was purified from CSs of PAO1 and MW1, respectively. Previously, we constructed a system for the expression of His-tagged PIV in *P*. *aeruginosa* using the pQF21C-PIV plasmid coupled with PAO-T7 strain^11^. To overexpress the same His-tagged PIV in MW1 background, we used the pQF21C-PIV coupled with PAO-T7-MW1 that cannot produce QS signals like MW1 (Table S1). Two His-tagged PIVs were purified from PAO-T7 (P-PIV) and PAO-T7-MW1 (M-PIV) using the Ni-NTA affinity chromatography (Fig. 2A). Both M-PIV and P-PIV contained PIV of the same size (26 kDa). However, a 22 kDa protein was always co-purified with M-PIV, whereas this protein was never co-purified with P-PIV (Fig. 2A). It was hard to remove the protein from the purified PIV fraction in size-exclusion chromatography, indicating that it tightly binds to PIV (data not shown). When we measured the activity of P-PIV and M-PIV for comparison, M-PIV had still severely reduced activity (Fig. 2B). Consistently, the virulence of M-PIV to *T*. *molitor* larvae was also much attenuated, compared with P-PIV (Fig. 2C,D,E).

We thought of two possibilities for the reduction of PIV activity in M-PIV; 1) any internal modification of PIV occurring only in M-PIV or 2) inhibition by the co-purified protein. In the first hypothesis, the PIV activity is inhibited by the internal modification and the 22 kDa protein should bind only to M-PIV, discriminating the modified structure. In the second hypothesis, the 22 kDa protein can bind to and inhibit PIV commonly, but it should exist only in QS-negative CS. To find out any difference in size, cleavage site, or post-translational modification of PIV, we performed N-terminal sequencing, LC-MS/MS analysis with the PIV bands in P-PIV and M-PIV, but all showed the same results, suggesting that two PIV bands contain actually same proteins (data not shown).

**Figure 3:**
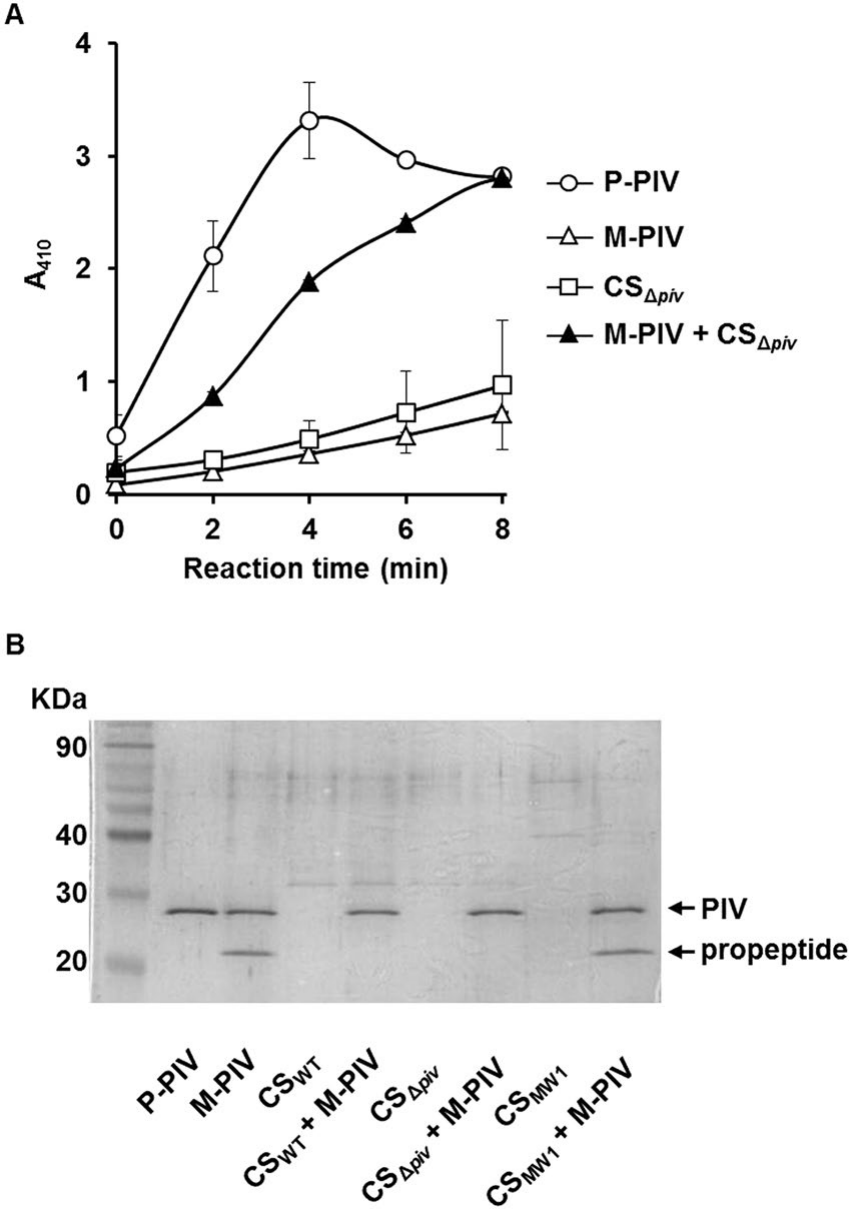
Activity restoration of M-PIV and propeptide degradation by CS from QS-positive strain. (**A**) 100 ng of M-PIV was mixed with 2 μl of 10 times-concentrated CS from Δ*piv* (CS_Δ*piv*_) and the PIV activity was promptly measured without incubation. As controls, same amount of M-PIV, P-PIV, and CS_Δ*piv*_ were measured for the PIV activity in the same condition. (**B**) CS_Δ*piv*_ was mixed with 400 ng of M-PIV in 25 μl reaction volume and incubated at room temperature for 30 min. For a control, the CSs from wild type (CS_WT_) or MW1 (CS_MW1_) were mixed and incubated with M-PIV in the same condition. All reactions were applied to SDS-PAGE and visualized by Coomassie staining. The propeptide and PIV bands are indicated. This gel picture was cropped and the full-length gel picture is presented in Fig. S4A.

In the second hypothesis, a QS-dependent factor in wild type CS should eliminate the 22 kDa protein to relieve the inhibition. To test this possibility, the CS of Δ*piv* strain that is QS-positive but has no PIV activity was prepared (CS_Δ*piv*_) and added to M-PIV. The CS of Δ*piv* can supply the QS-dependent extracellular factors without increase of the PIV activity. When the activity of M-PIV was measured, each M-PIV and CS_Δ*piv*_ showed lower activity than P-PIV, whereas the M-PIV mixed with CS_Δ*piv*_ showed comparable activity to P-PIV (Fig. 3A). This result demonstrated that a QS-dependently produced factor can restore the activity of M-PIV extracellularly.

### PIV is inhibited by its propeptide that is degraded by QS-dependent extracellular factor for the activation of PIV

We got a hint from the maturation mechanism of LasB (elastase), another QS-dependent extracellular protease of *P*. *aeruginosa*. LasB is initially expressed as 53.6 kDa prepro-LasB containing a signal sequence and propeptide at N-terminus that are removed during secretion to generate a periplasmic 33 kDa mature LasB^18, 19^. The cleaved 18 kDa propeptide binds to and inhibit the mature LasB in extracellular space^20^. There was no conspicuous homology between amino acid sequences of the propeptides of PIV and LasB. Nevertheless, we thought that the mechanism might be similar and the 22 kDa protein might be the propeptide of PIV. N-terminal sequencing for the 22 kDa protein showed the sequence of APGAS that is well matched to the known N-terminal amino acid sequence of the PIV propeptide (Fig. S1)^17^. This result implied that the propeptide of PIV might exist as a complex with PIV in the M-PIV fraction by its high affinity to PIV and the PIV activity would be restored if the propeptide is degraded as in QS-positive wild type. In order to prove this, the M-PIV containing the propeptide of PIV was mixed with the CSs of wild type or Δ*piv*, the QS positive strain. As we expected, the propeptide band disappeared with CS_WT_ and CS_Δ*piv*_, but did not with CS_MW1_ (Fig. 3B). These results demonstrate that PIV can be inhibited by binding of its propeptide, but in wild type, the propeptide is usually degraded by QS-dependent extracellular factor to activate PIV.

### The QS-dependent factor responsible for the propeptide degradation is LasB

**Figure 4:**
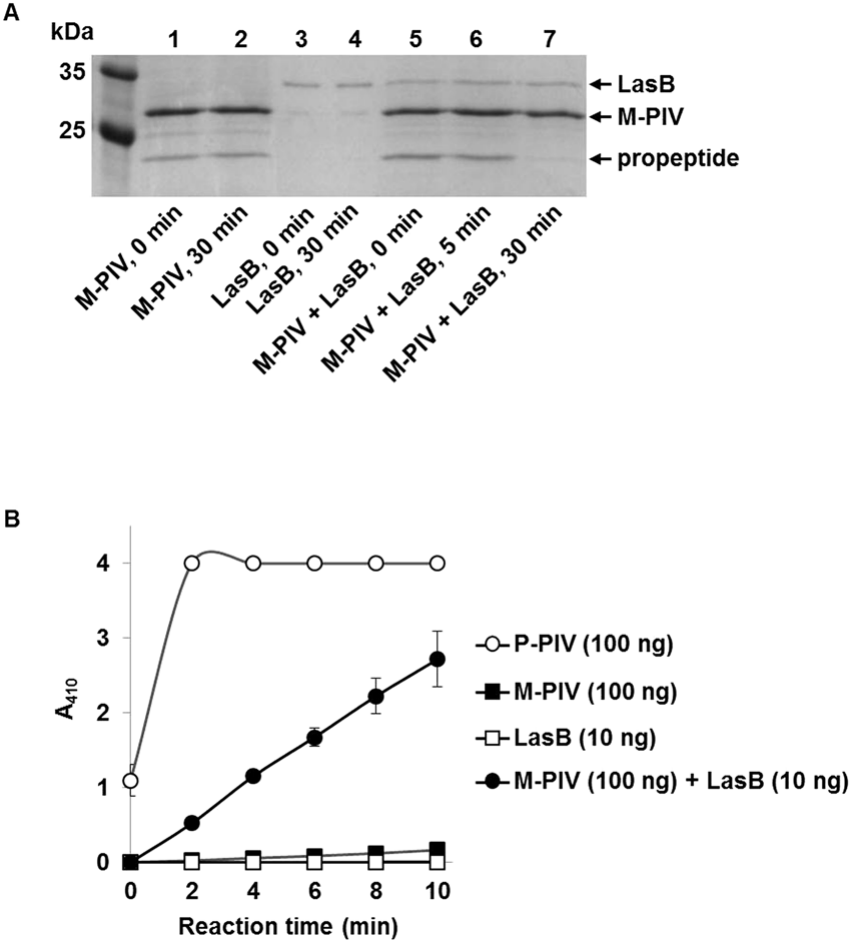
Propeptide degradation and activity restoration of M-PIV by LasB. (**A**) The purified 100 ng of LasB was mixed with 800 ng of M-PIV in 25 μl reaction volume and incubated at room temperature (RT) for 30 min. For control, the purified LasB and M-PIV were separately incubated in the same condition. All fractions were applied to 12% SDS-PAGE and visualized by Coomassie staining. Lane 1–2, M-PIV alone with no or 30 min incubation; lane 3–4, LasB alone with no or 30 min incubation; lane 5–7, M-PIV + LasB with no, 5, or 30 min incubation. This gel picture was cropped and the full-length gel picture is presented in supplementary data (Fig. S4B). (**B**) The PIV activities of P-PIV, M-PIV, LasB, and M-PIV + LasB were measured by chromogenic substrate. The amount of the purified proteins used in each reaction is indicated. The activity was measured promptly after adding the proteins without incubation.

Since the propeptide of PIV is degraded extracellularly in a QS-dependent manner, the factor responsible for the propeptide degradation was thought to be a QS-dependently produced extracellular protease. A QS-transcriptome analysis of *P*. *aeruginosa* revealed eight QS-dependently expressed extracellular proteases (LasA, LasB, PIV, PA0355, PA1249, PA2939, PA3535, and PA4171)^7^. Except PIV, seven proteases were overexpressed on pJN105-based plasmids in MW1 by arabinose induction (Table S1), and the CSs from the protease-overexpressing MW1 were prepared. When the CSs were added to M-PIV containing propeptide and incubated at room temperature for 30 min, only the LasB-overexpressing CS was able to degrade the propeptide (Fig. S3). To confirm this, purified LasB was mixed with M-PIV and incubated in the same condition. The result showed rapid degradation of the propeptide of PIV (Fig. 4A). In addition, the M-PIV activity was restored by addition of purified LasB, in which 10 ng of purified LasB was able to significantly restore the PIV activity of 100 ng M-PIV (Fig. 4B). These results demonstrated that PIV is activated in extracellular space through the degradation of its propeptide by LasB, a QS-dependent extracellular protease.

### The propeptide binds to PIV and inhibits the PIV activity

**Figure 5:**
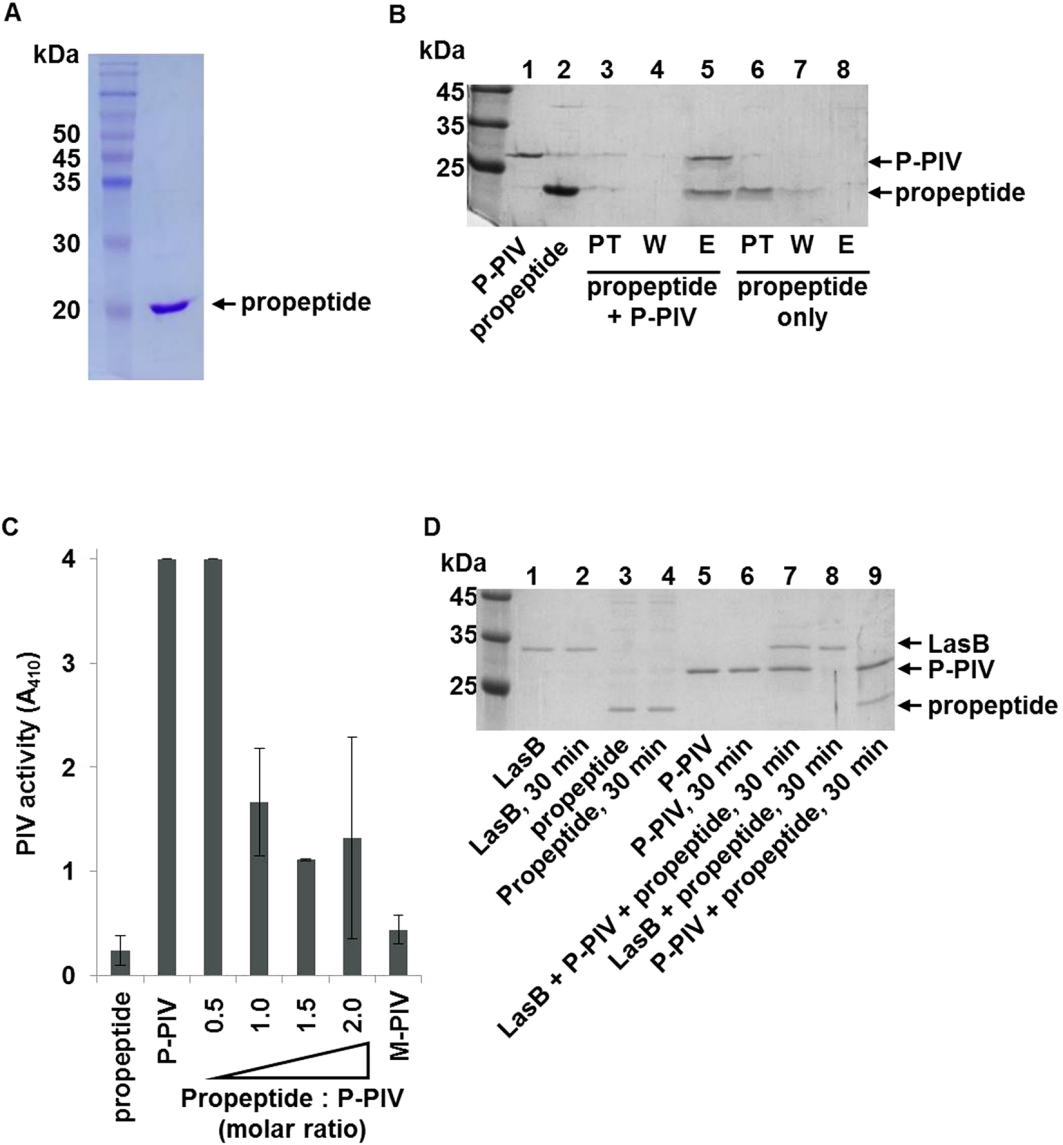
Binding of propeptide to PIV and inhibition of PIV activity. The propeptide of PIV was purified from *E*. *coli* (**A**). (**B**) 1 μg of the purified propeptide was mixed with 1 μg of P-PIV in 25 μl reaction volume and incubated at room temperature for 10 min. The mixture was loaded to Ni-NTA agarose affinity column, washed twice with 5 mM imidazole-containing washing buffer, and eluted by 200 mM imidazole-containing elution buffer (lanes 3–5). Same amount of the purified propeptide was applied to Ni-NTA agarose affinity column in the same manner (lanes 6–8). PT, W, and E indicate fractions of pass through, wash, and elute, respectively. The purified propeptide of PIV and P-PIV were loaded as control (lane 1, 2). This gel picture was cropped and the full-length gel picture is presented in supplementary data (Fig. S4C). (**C**) The purified propeptide was mixed with 100 ng of P-PIV at the indicated molar ratio and incubated in room temperature for 10 min. The PIV activity was then measured by chromogenic substrate. As controls, 160 ng of propeptide, 100 ng of P-PIV, or 100 ng of M-PIV were separately measured for the PIV activity for comparison. (**D**) 500 ng of the purified LasB, 1 μg of propeptide, and 1 μg of P-PIV were mixed in various combination and incubated at room temperature for 30 min. The proteins were analyzed in 12% SDS-PAGE and visualized by Coomassie staining. To know stability, each protein was directly applied to SDS-PAGE without incubation (lanes 1, 3, 5). This gel picture was cropped and the full-length gel picture is presented in supplementary data (Fig. S4D).

In order to clearly prove the binding of propeptide to PIV, we overexpressed and purified the propeptide of PIV from *E*. *coli* (Fig. 5A). When the purified propeptide was mixed with P-PIV that is His-tagged active PIV and loaded on Ni-NTA agarose affinity column, the propeptide was co-eluted with P-PIV, showing its binding to PIV (Fig. 5B, lane 3–5). Since the propeptide does not have His-tag, it alone was not able to bind to Ni-NTA agarose resin (Fig. 5B, lane 6–7). This result explains the co-purification of propeptide in M-PIV. When we measured the PIV activity with the addition of propeptide to P-PIV, the PIV activity was inhibited (Fig. 5C), demonstrating that the binding of propeptide to P-PIV inhibited the PIV activity. Finally, when we mixed the purified LasB with P-PIV and propeptide, LasB specifically degraded only the propeptide and did not impair P-PIV (Fig. 5D). All these results consistently demonstrate that the propeptide of PIV binds to PIV and inhibits the PIV activity, and LasB relieves this inhibition by specifically degrading the propeptide.

## Discussion

**Figure 6:**
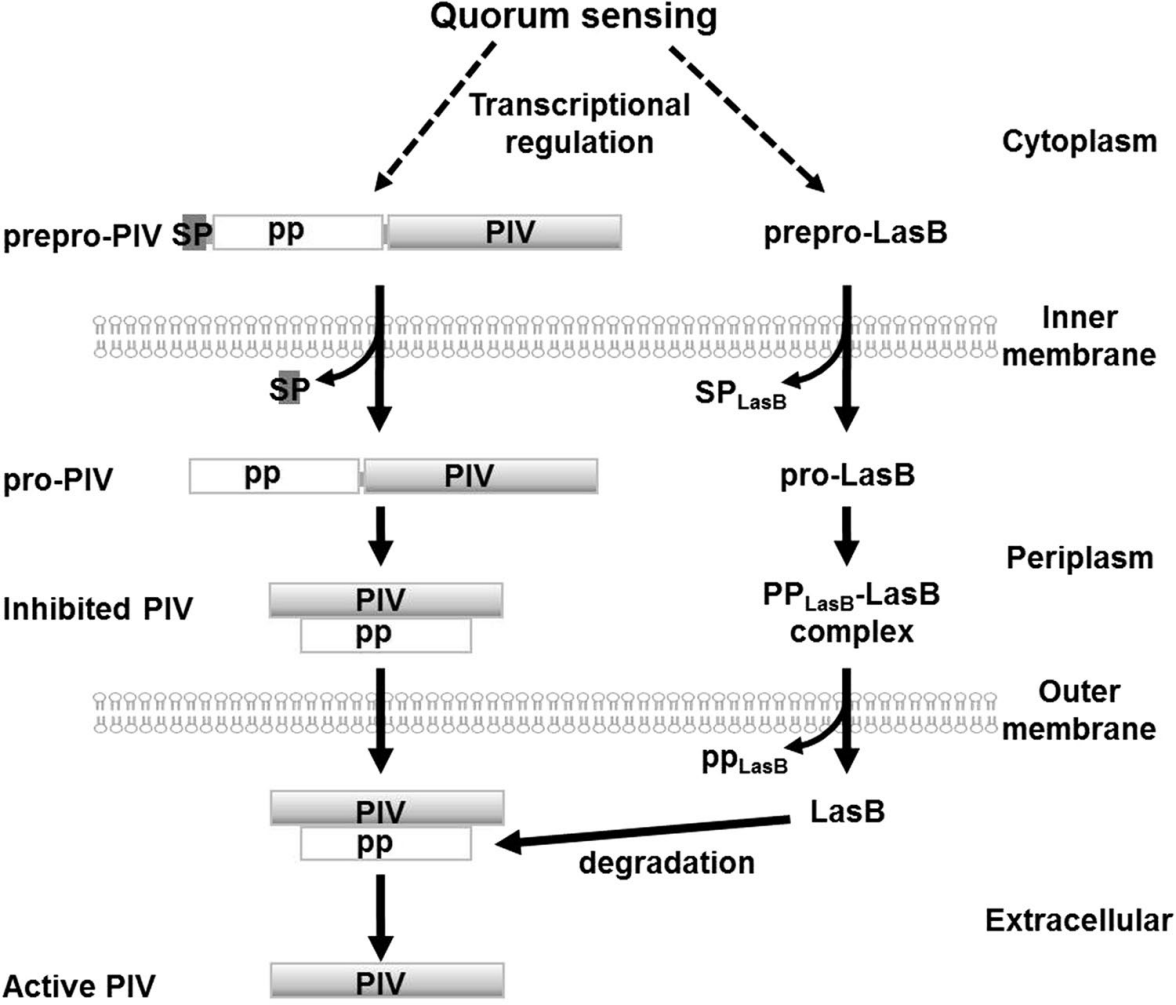
QS-mediated post-secretional activation of PIV. The initial expression of both PIV and LasB is positively regulated by QS. The prepro-PIV in cytoplasm is processed twice from N-terminus during secretion to release signal peptide, propeptide, and mature PIV in extracellular space. The mature PIV is inhibited by the propeptide binding. The mature LasB then degrades the propeptide to release activate PIV. LasB is also processed in a similar manner and inhibited by its propeptide (PP_LasB_). The processing of pro-LasB to mature LasB is achieved by auto-cleavage. SP, signal peptide; PP, propeptide.

PIV that is tightly regulated by QS system plays a crucial role in the *Pseudomonas* infections by degrading crucial proteins in host immune system like complement and IgG^2^, host structural proteins like elastin, and the iron-binding proteins like lactoferrin and transferrin^21^. In insect, it degrades innate immune components^11^. So far, the QS-dependence of PIV has been thought to be due to the transcriptional regulation of *piv* gene by the QS system. However, in this study, we newly found that there is additional QS-mediated regulation on the PIV activity, which is exerted after secretion of PIV. Our suggestion is summarized in Fig. 6. Although the expression of PIV is induced by QS at the transcription level, its activity is still restrained until it is completely secreted into extracellular space, because the propeptide cleaved from pro-PIV holds PIV inactive in periplasm. This type of inhibition by the propeptide may be important to prevent the undesired degradation of periplasmic proteins by the PIV activity, because proteases such as PIV can be potentially harmful to bacterial cells producing them, as well as virulent to host. Therefore, the role of propeptide can be thought of as a “safety pin of a hand grenade”.

Similar inhibition by own propeptides has been reported in the maturation of LasB and LasA of *P*. *aeruginosa*. As illustrated in Fig. 6, LasB is also processed in a similar manner and inhibited by its propeptide. LasB is initially expressed as 53.6 kDa full length prepro-enzyme (prepro-LasB) in cytoplasm and further processed like PIV during secretion. However, the cleaved propeptide of LasB still attaches to mature LasB and inhibits its activity^20^. The propeptide dissociates to activate LasB and is degraded in the extracellular space. Like PIV and LasB, LasA is expressed as a 40 kDa prepro-enzyme in cytoplasm and processed twice from N-terminus during secretion into periplasm. After first processing to remove the signal peptide, the pro-form of LasA is exported to extracellular environment without cleavage of propeptide^22^. pro-LasA is finally activated when its propeptide is cleaved and degraded.

An important common mechanism for the extracellular activation of LasB, LasA, and PIV is the degradation of their own propeptide. It is not fully elucidated how the propeptide of LasB is degraded, but it has been presumed to be degraded by LasB itself  ^22^. It has been proposed that the removal and degradation of the LasA propeptide is performed by multiple extracellular proteases, LasB, LysC (aspartokinase), and PIV^22^. In this study, we found that the PIV propeptide is degraded by LasB. In all mechanisms, LasB is mainly responsible for triggering the extracellular activation of these proteases. In that the expression of LasB is regulated by QS system in *P*. *aeruginosa*, we think that QS substantially dominates the activity of the extracellular proteases. This idea is supported by our observation that the PIV activity is not increased by artificial overexpression of PIV in QS-negative strain.

LasB is an elastase of *P*. *aeruginosa*. Elastase is a very common protease in nature and produced by many organisms including human. So, the propeptide-inhibited extracellular proteases may be activated by elastases of host in infection sites. In addition, we note that although LasB is a main factor to degrade the propeptides, it may not be the only factor to do it in *P*. *aeruginosa*. Actually, PIV was originally characterized in an elastase-deficient strain (PA103-29) that has high caseinase activity^2, 23^. This means that the elastase deficiency of the strain was compensated with increase of other protease activity, which can activate PIV instead of elastase. Nonetheless, we think that even with such alternative proteases, it is under the control of QS, because the overexpressed PIV is not activated in the QS mutant.

## Experimental Procedures

### Bacterial strains and culture conditions

Bacterial strains and plasmids used in this study are listed in Table S1. *P*. *aeruginosa* strains are grown at 37 °C in Luria-Bertani (LB; yeast 5 g/L, bacto-tryptone 10 g/L, NaCl 5 g/L) medium with vigorous shaking. Growth was measured by optical density at 600 nm (OD_600_). For the cultivation of plasmid-carrying *P*. *aeruginosa* strains, the plasmids were introduced into PAO1 and MW1 by transformation with antibiotic selection. The transformed cells harboring each plasmid were then grown with antibiotics as above. To induce the protein expression, arabinose (for pJN105-based plasmids) or IPTG (Isopropyl-1-thio-β-D-thiogalactopyranoside, for pQF21c-PIV and pET16b-pro) were added at 0.2% and 0.1 mM, respectively. Antibiotics were used at the following concentrations: carbenicillin, 150 μg/ml (for *P*. *aeruginosa*); gentamicin, 50 μg/ml (for *P*. *aeruginosa*); tetracycline, 60 μg/ml (for *P*. *aeruginosa*).

### Preparation of the culture supernatants (CSs)

For the preparation of CSs, cells were grown in 5 ml of LB with vigorous shaking at 37 °C overnight and diluted 1:100 into fresh 5 ml of LB broth for main culture with 0.2% arabinose. After further cultivation with vigorous shaking at 37 °C overnight, cells were removed by centrifugation at 13,000 rpm for 2 min at 4 °C and the supernatants were taken to be filtered through 0.22 μm syringe filter (Satorious). The CSs were concentrated 10 times in 10 kDa cut-off centricon (Vivaspin^®^, Satorious).

### Virulence assay with *Tenebrio molitor* larvae

*T*. *molitor* were maintained in a terrarium with wheat bran on a laboratory bench and virulence assay using *T*. *molitor* larvae was performed as described elsewhere^24^. Briefly, 5 μl of the CSs were carefully injected into *T*. *molitor* larvae using syringe. As controls, same volume of insect saline (130 mM NaCl, 5 mM KCl, 1 mM CaCl_2_) and the CS prepared from cells harboring empty pJN105 (vector control) were injected. The larvae were incubated in petri dishes at 30 °C in a dark place and observed to count the live/dead and melanization for 4 days. The dead larvae turn black as a result of melanization and do not respond to touch.

### Overexpression and purification of Protease IV (PIV) and LasB

The pQF21c-PIV plasmid^11^ was introduced into *P*. *aeruginosa* PAO-T7 (for the P-PIV purification) or PAO-T7-MW1 (for the M-PIV purification) by transformation (Table S1). The resulting cells harboring pQF21c-PIV were inoculated to 1 L of LB broth with 150 μg/ml cabenicillin and cultivated at 37 °C with vigorous shaking up to OD_600_ = 0.5. Then, IPTG (Isopropyl-1-thio-β-D-galactopyranoside) was added to 0.1 mM. After 16-hour further cultivation, cells were removed by centrifugation at 12,000 rpm for 30 min at 4 °C and the supernatant was taken to be filtered through 0.22 μm filter. The resulting cell-free supernatant was applied to the binding buffer (20 mM Tris-HCl (pH 7.9), 500 mM NaCl)–equilibrated Ni-NTA agarose affinity resin (Novagen) in column chromatography. After washing with binding buffer, the bound proteins were eluted by imidazole-containing elution buffer (20 mM Tris-HCl (pH 7.9), 500 mM NaCl, 5–500 mM imidazole). The fractions containing His-tagged PIV were pooled and dialyzed in storage buffer (50 mM Tris-HCl (pH 7.5), 50 mM NaCl, 50 mM KCl and 30% glycerol). The purified His-tagged PIV was aliquoted and stored at −80 °C. For the LasB purification, LasB-overexpressing plasmid (pSP201, Table S1) was introduced into the PAO1 and the transformed cells were cultivated in LB medium at 37 °C with vigorous shaking. Arabinose was added at 0.8% to induce LasB for 16 hours. Cells were then removed by centrifugation and the culture supernatant was taken. LasB was precipitated by slow addition of ammonium sulfate and pelleted by centrifugation. The pellet was dissolved in 50 mM Tris-HCl (pH 8.0) and the remained salt was completely removed by dialysis. This LasB-containing solution was applied to DEAE sepharose column chromatography and eluted by NaCl gradient. The fractions containing pure LasB were collected and dialyzed in the storage buffer. The purified LasB was aliquoted and stored at −80 °C.

### Activity assay of PIV

PIV activity was determined by using chromogenic substrate (N-(p-Tosyl)-Gly-Pro-Lys-4-nitroanilide acetate salt; Sigma), as described elsewhere^2, 17^. Since PIV, a lysyl endopeptidase cleaves on the carboxyl side of lysine residues, N-p-Tosyl-Gly-Pro-Lys-4-nitroanilide is cleaved by PIV, releasing nitroanilide that can be spectrophotometrically measured. The indicated amount of purified proteins or CSs prepared from various strains was added to 100 μl reaction buffer (50 mM Tris-HCl (pH 8.0)) containing 200 μM chromogenic substrate. The reaction was incubated at 37 °C and the absorbance was measured at 410 nm (A_410_) every 2 min for 10 min.

### Overexpression and purification of propeptide

The internal region of *piv* gene including signal peptide and propeptide of PIV (from 1^st^ to 211^th^ amino acid, Fig. S1) was amplified by PCR and ligated into the *Nde*I and *Bam*HI-digested pET16b plasmid to make pET16b-pro (Table S1). The primers used in PCR amplification were 5′-CGAACATATGCATAAGAGAACGTAC-3′ and 5′-CGCGGGATCCTCACTTGTACAGGGAGTCGG-3′ (*Nde*I and *Bam*HI sites are underlined). *E*. *coli* BL21 (DE3) harboring pET16b-pro was growth at 37 °C in 2 L of LB broth containing 100 μg/ml ampicillin. At OD_600_ = 0.5, IPTG was added at 0.1 mM to induce the propeptide expression and cells were further cultivated overnight at 16 °C with vigorous shaking. Cells were harvested by centrifugation for 20 min at 4 °C and lysed by sonication in binding buffer (20 mM Tris-HCl, 500 mM NaCl, and 10 mM imidazole, pH 8.0). The soluble fraction was applied to Ni-NTA column (Invitrigen) and the bound proteins were washed and eluted by increasing concentrations of imidazole. Fractions containing pro-peptide were pooled, and the N-terminal part including His-tag and signal peptide was cleaved off. The resulting propeptide was dialyzed in 50 mM Tris-HCl buffer (pH 8.0) and loaded on Q sepharose column (GE Healthcare). The protein was eluted with NaCl-gradient in 50 mM Tris-HCl buffer (pH 8.0). The propeptide-containing fractions was pooled and further purified by size exclusion chromatography with HiLoad 16/60 Superdex 200 gel filtration column (GE Healthcare). Fractions containing pure propeptide were pooled, dialyzed in storage buffer, and stored at −80 °C. The removal of His-tag and signal peptide, and intact N-terminus of propeptide was confirmed by N-terminal sequencing.

### Statistical analysis

In order to ensure the significance of the results in the virulence analyses, the data were statistically analyzed using *t*-test (two-sample assuming equal variances) in MS office Excel (Microsoft, USA). If the *P*-value was lower than 0.01, it was considered significant.

## Electronic supplementary material

Supplementary Dataset
