# Supplementary material for: Post-secretional activation of Protease IV by quorum sensing in Pseudomonas aeruginosa
Source: Sci Rep. 2017 Jun 30;7:4416. doi: 10.1038/s41598-017-03733-6 (PMC5493658; doi:10.1038/s41598-017-03733-6)
Supplement: Supplementary file 1 — Supplementary Dataset [file 41598_2017_3733_MOESM1_ESM.doc]

**Post-secretional activation of Protease IV by quorum sensing in *Pseudomonas aeruginosa***

**Jungmin Oh, Xi-Hui Li, Soo-Kyoung Kim, and Joon-Hee Lee***

**Department of Pharmacy, College of Pharmacy, Pusan National University, Busan, 609-735, South Korea**

**Table S**1. Bacterial strains and plasmids used in this study

| **Name** | **Description** | **References** |
| --- | --- | --- |
| ***P. aeruginosa*** |  |  |
| PAO1 | Wild type *P. aeruginosa* | 1 |
| MW1 | *lasI^-^,rhlI^-^* double mutant of PAO1 | 2 |
| DH0001 | *piv^-^* mutant of PAO1, Tc^R^ | 3 |
| PAO-T7 | T7 RNAP*-expressing PAO1 | 4 |
| PAO-T7-MW1 | T7 RNAP-expressing MW1 | 5 |
| ***E. coli*** |  |  |
| DH5α | *supE44*Δ*lacU*169(80*lacZ*ΔM15)*hsdR17* *recA1* *gyrA96thi-1 relA1* | Lab. collection |
| BL21(DE3) | F^-^*omp*T*hsd*S_B_ (r_B_^-^ m_B_^-^) *dcm* *gal*λ (DE3) | Lab. collection |
| **Plasmids** |  |  |
| pJN105 | *araC*-pBAD cassette cloned in pBBR1MCS, Gm^R^ | 6 |
| pSP101 | PA1871 (*lasA*) in pJN105, Gm^R^ | 3 |
| pSP201 | PA3724 (*lasB*) in pJN105, Gm^R^ | 3 |
| pSP301 | PA4175 (*piv*) in pJN105, Gm^R^ | 3 |
| pSP401 | PA2939 in pJN105, Gm^R^ | 3 |
| pKS101 | PA0355 in pJN105, Gm^R^ | 3 |
| pHP101 | PA1249 in pJN105, Gm^R^ | 3 |
| pHP201 | PA3535 in pJN105, Gm^R^ | 3 |
| pHP301 | PA4171 in pJN105, Gm^R^ | 3 |
| pQF21c | Modified pET21c that has broad-host-range replication origin, Ori_1600_ and pMB1, Cb^R^ | 3 |
| pQF21c-PIV | *piv* in pQF21c, Cb^R^ | 3 |
| pET16b | Overexpression plasmid for His-tagged proteins, Ap^R^ | Novagen |
| pET16b-pro | Propeptide-coding region of PIV in pET16b, Ap^R^ | This work |

RNAP, RNA polymerase; Tc, Tetracycline; Gm, Gentamicin; Cb, Carbenicillin; Ap, Ampicillin

**
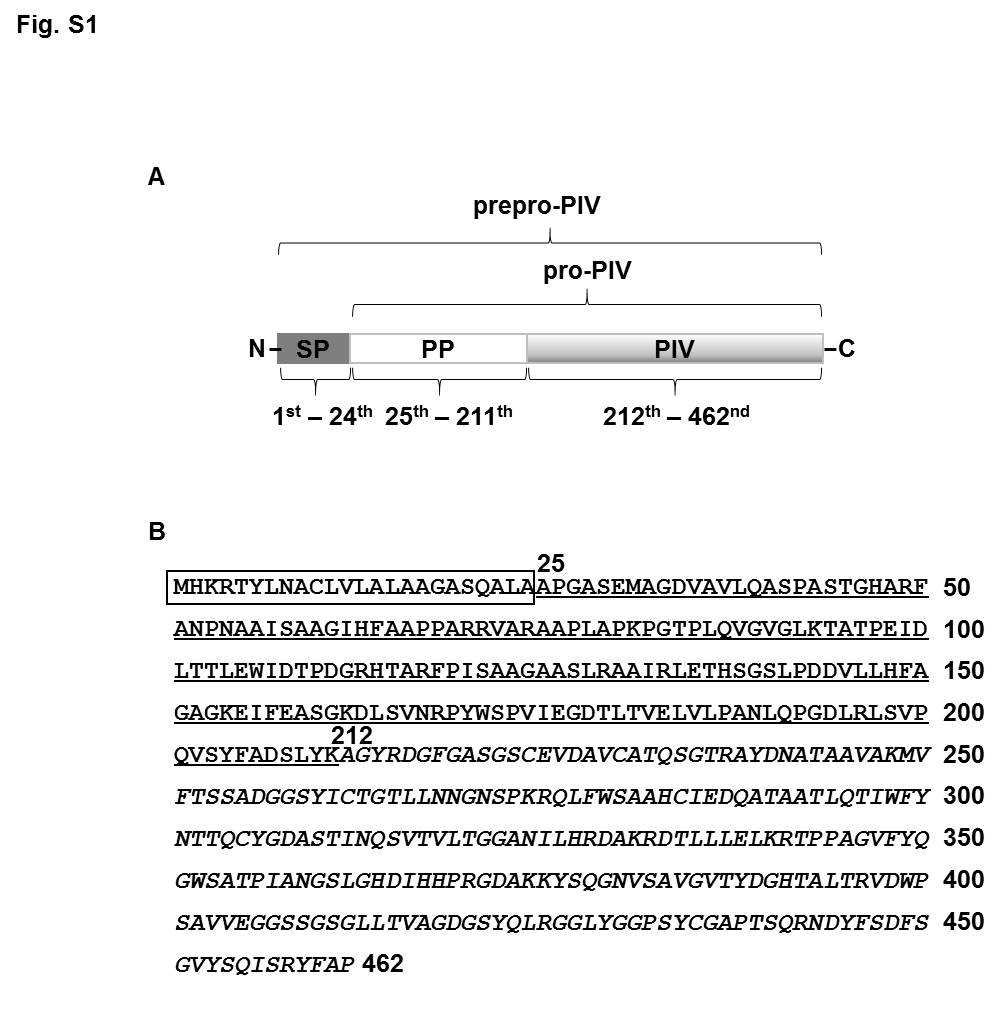
**

**Fig. S1. The structure of full length PIV (prepro-PIV)** A, the structure of full length PIV (prepro-PIV) that is initially expressed from *piv* gene in cytoplasm is illustrated. prepro-PIV is composed of 462 amino acids in which signal peptide (SP) locates at 1^st^ - 24^th^ amino acids, propeptide (PP), 25^th^ – 211^th^, and PIV (mature PIV), 212^th^ – 462^nd^. B shows full amino acid sequence of prepro-PIV in which SP is boxed, PP, underlined, and PIV, italicized.

**
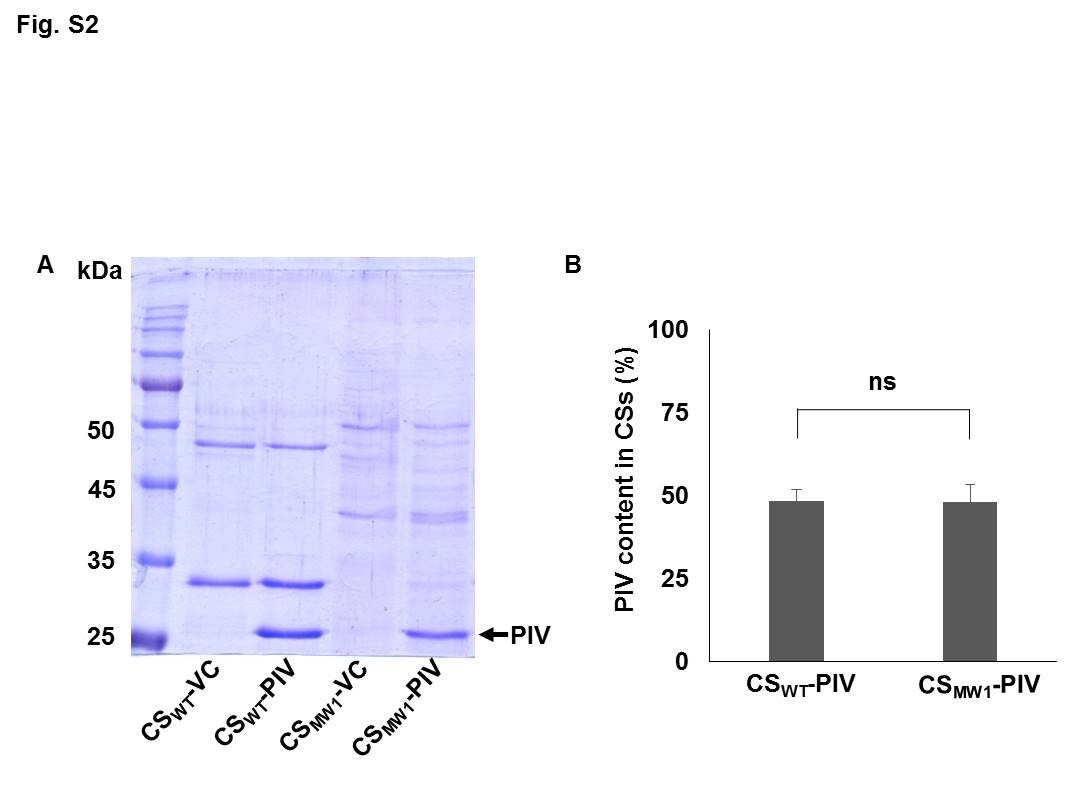
**

**Fig. S2. The amount of PIV in CS_WT_-PIV and CS_MW1_-PIV for comparison** CS_WT_-PIV and CS_MW1_-PIV were loaded to SDS-PAGE and visualized by Coomassie staining (A). The PIVs are indicated. The content of PIV in two CSs was quantified by Image J program (B). ns, not significant.

**
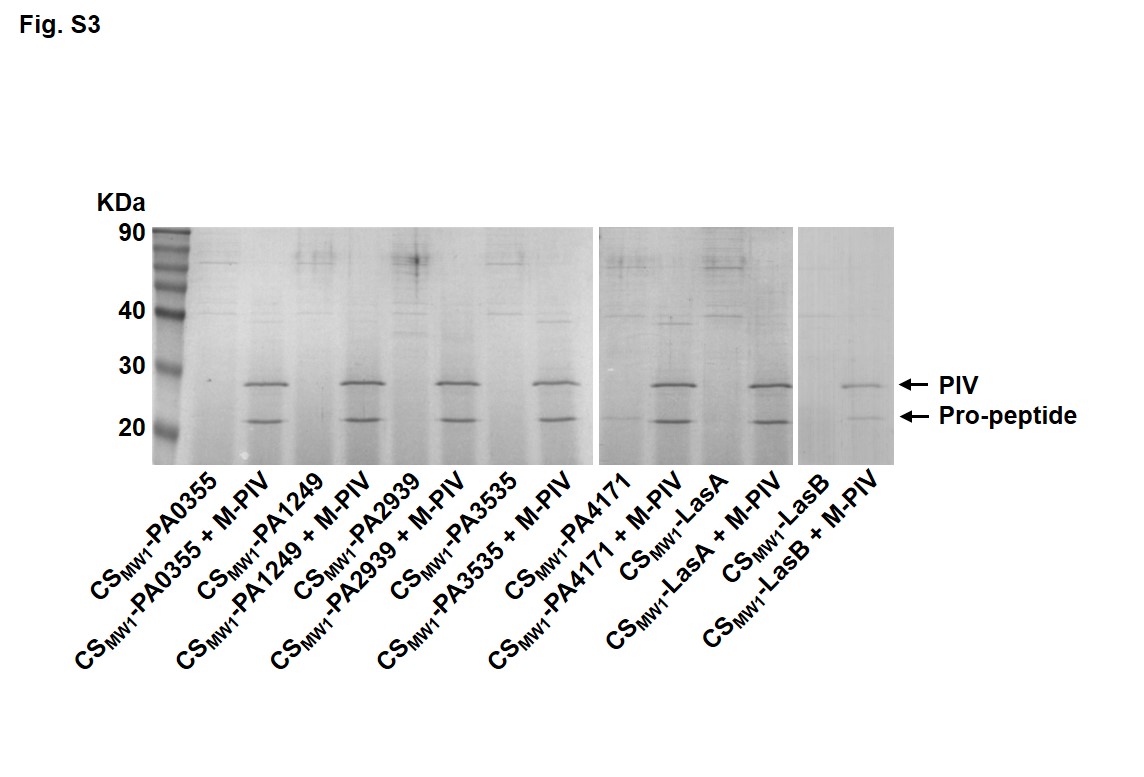
**

**Fig. S3. Screening of the QS-dependent factor responsible for the propeptide degradation** The CSs were prepared from the MW1 cells expressing the QS-dependently expressed extracellular proteases. They were mixed with 400 ng of M-PIV containing propeptide and incubated at room temperature for 30 min. All reactions were applied to SDS-PAGE and visualized by Coomassie staining. The propeptide and PIV bands are indicated.


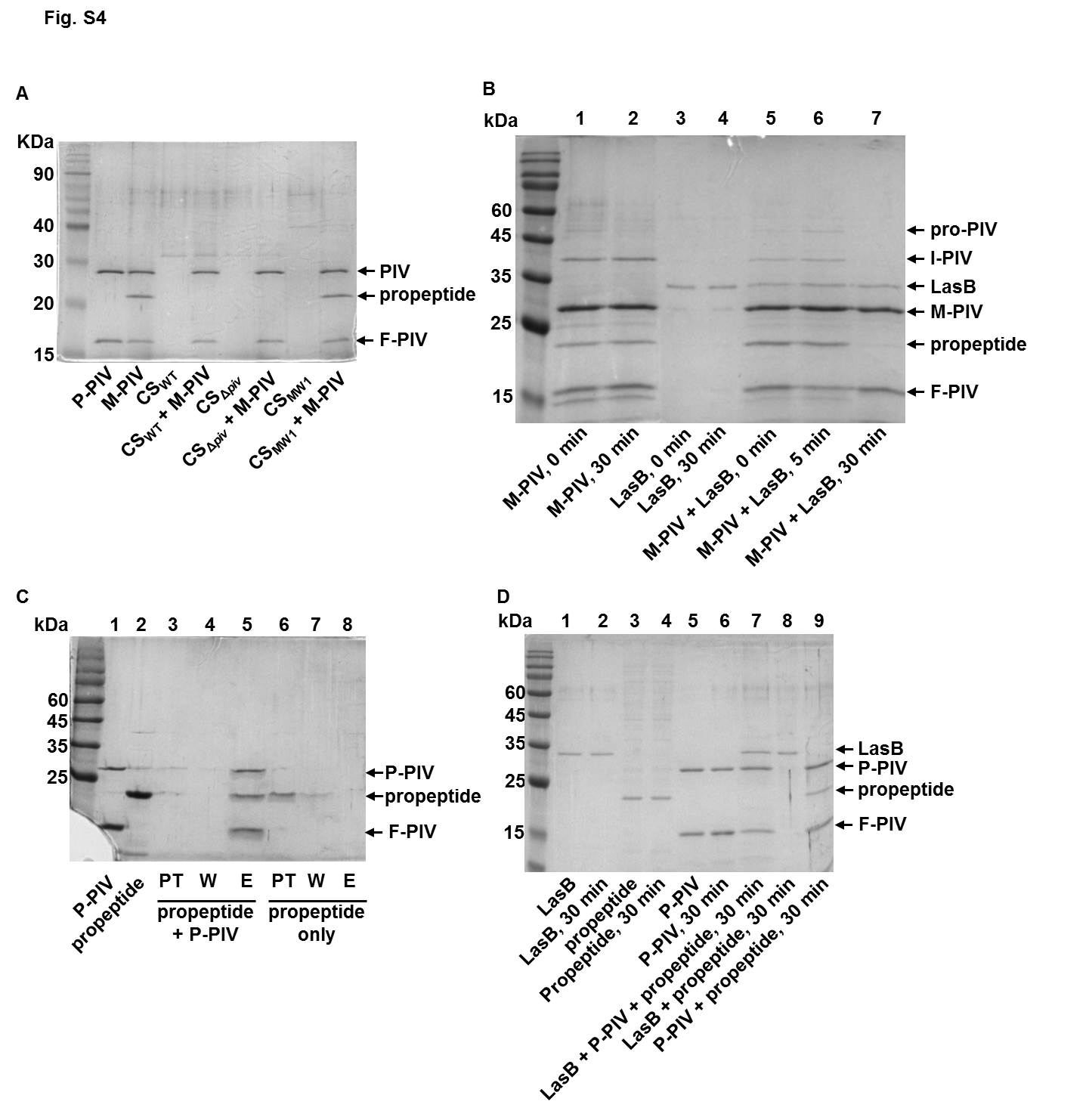


**Fig. S4. Full-length gel images** A, full-length gel picture of Fig. 3B. An extra protein band (17 kDa) is indicated as F-PIV. F-PIV is a further-processed form of PIV that is still active. Further degradation of PIV to F-PIV has been also observed by other group ^7^. B, full-length gel picture of Fig. 4A. Extra protein bands for pro-PIV (45 kDa), I-PIV (40 kDa), and F-PIV (17 kDa) are indicated. Pro-PIV is non-processed form of PIV and I-PIV may be a processing intermediate of PIV. I-PIV was sometimes co-purified with PIV, but not always, and similar processing intermediate has been observed in the LasA maturation ^8^. C, full-length gel picture of Fig. 5B. An extra protein band for F-PIV is indicated. D, full-length gel picture of Fig. 5D. F-PIV is also indicated.

**References for supplementary data**

1 Pearson, J. P., Pesci, E. C. & Iglewski, B. H. Roles of Pseudomonas aeruginosa las and rhl quorum-sensing systems in control of elastase and rhamnolipid biosynthesis genes. *Journal of bacteriology* **179**, 5756-5767 (1997).

2 Whiteley, M., Lee, K. M. & Greenberg, E. P. Identification of genes controlled by quorum sensing in *Pseudomonas aeruginosa*. *Proc. Natl. Acad. Sci. USA* **96**, 13904-13909 (1999).

3 Park, S. J. *et al.* Protease IV, a quorum sensing-dependent protease of Pseudomonas aeruginosa modulates insect innate immunity. *Molecular microbiology* **94**, 1298-1314, doi:10.1111/mmi.12830 (2014).

4 Hoang, T. T., Kutchma, A. J., Becher, A. & Schweizer, H. P. Integration-proficient plasmids for *Pseudomonas aeruginosa*: site-specific integration and use for engineering of reporter and expression strains. *Plasmid* **43**, 59-72 (2000).

5 Hirakawa, H. *et al.* Activity of the Rhodopseudomonas palustris p-coumaroyl-homoserine lactone-responsive transcription factor RpaR. *Journal of bacteriology* **193**, 2598-2607, doi:10.1128/JB.01479-10 (2011).

6 Newman, J. R. & Fuqua, C. Broad-host-range expression vectors that carry the L-arabinose-inducible *Escherichia coli araBAD* promoter and the araC regulator. *Gene* **227**, 197-203 (1999).

7 Engel, L. S., Hill, J. M., Caballero, A. R., Green, L. C. & O'Callaghan, R. J. Protease IV, a unique extracellular protease and virulence factor from Pseudomonas aeruginosa. *The Journal of biological chemistry* **273**, 16792-16797 (1998).

8 Kessler, E., Safrin, M., Gustin, J. K. & Ohman, D. E. Elastase and the LasA protease of Pseudomonas aeruginosa are secreted with their propeptides. *The Journal of biological chemistry* **273**, 30225-30231 (1998).
